# An Overview of Diode Laser-Assisted Oral Surgery

**DOI:** 10.7759/cureus.9297

**Published:** 2020-07-20

**Authors:** Domenico De Falco, Daniela Di Venere, Eugenio Maiorano

**Affiliations:** 1 Dentistry, University of Bari Aldo Moro, Bari, ITA; 2 Department of Emergency and Organ Transplantation, University of Bari Aldo Moro, Bari, ITA

**Keywords:** oral surgery, diode laser, photocoagulation, venous malformations

## Abstract

Among all lasers with generally accepted surgical capabilities, the diode laser is most commonly used for the surgical removal of proliferating lesions and the photocoagulation of venous malformations of the oral cavity. The laser provides several advantages for clinicians, including an absence of intraoperative bleeding and no need for stitches. The laser benefits patients because it reduces postoperative edema and pain, with fast mucosal restoration during healing by second intention. We report a case series to highlight the capabilities of the diode laser in oral surgery procedures along with our suggestions.

## Introduction

The main properties of the diode laser (DL) are its targeted selectivity for oxyhemoglobin, induction of photothermolysis, erythrocyte microagglutination, and vessel obliteration [1-3]. The practical result is essentially the capability to coagulate tissue during cutting as well as directly coagulate small and large venous malformations [3-6]. Other generally accepted advantages of DL surgery are the reduction in the amount of locoregional anesthesia that is required, reduction of postoperative edema and pain, no need to apply stitches, and fast mucosal restoration for second intention healing [7-9]. With all of these advantages, the DL is used in many oral surgical and nonsurgical applications: frenectomy/frenulotomy; gingival remodeling; removal of benign, potentially malignant, and malignant lesions; surgical and nonsurgical periodontal treatments including drug-related gingival overgrowth; and photocoagulation of venous malformations [3,4,6,8-11]. Moreover, the relatively low cost and the small dimensions of the medical device are certainly adjunctive reasons for its extensive use among clinicians in outpatient and hospital settings [2,6,11]. Herein, the authors report a case series to highlight the capabilities of DL in minor and major oral surgery.

## Case presentation

Case 1

**Figure 1:**
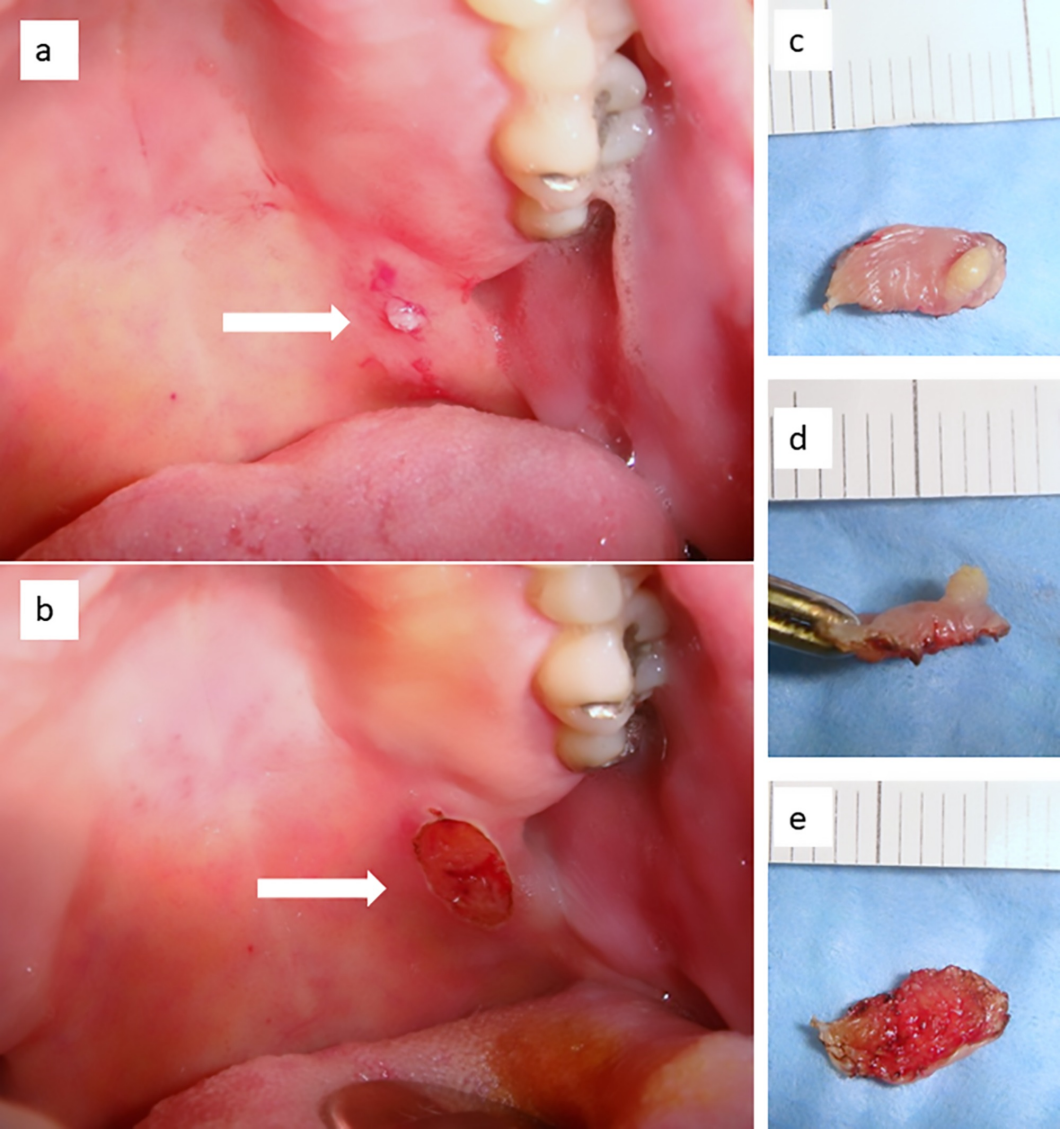
(a) Small nodular lesion within a larger white lesion of the posterior soft palate (b) surgically removed by diode laser with local infiltration of anesthesia and under conscious sedation; (c-e) the surgical specimen is well conserved and shows no sign of carbonization, which could affect histological readability

A small nodular lesion arose within a white lesion of the left posterior soft palate in a 45-year-old woman (Figure 1a). The lesion was painless but has been persistent for eight months. With the exclusion of a smoking habit, no other relevant data were reported in her medical history. Considering the anatomic site and the difficulty of a surgical approach, the lesion was removed via DL (wavelength 980 ± 10 nm; continuous wave; fiber of 320 microns; output energy 1 W) using local anesthesia and light conscious sedation for the patient (Figure 1b). Surgery was easy, bleeding was absent, and no postoperative complications occurred. The surgical specimen exhibited no alteration related to the thermal cut (Figures 1c-1e), thus leading to the histological diagnosis of mucocele associated with a leukoplakia without sign of dysplasia, and the latter was probably related to the smoking habit. The wound healed in 10 days, and there was no recurrence.

Case 2

**Figure 2:**
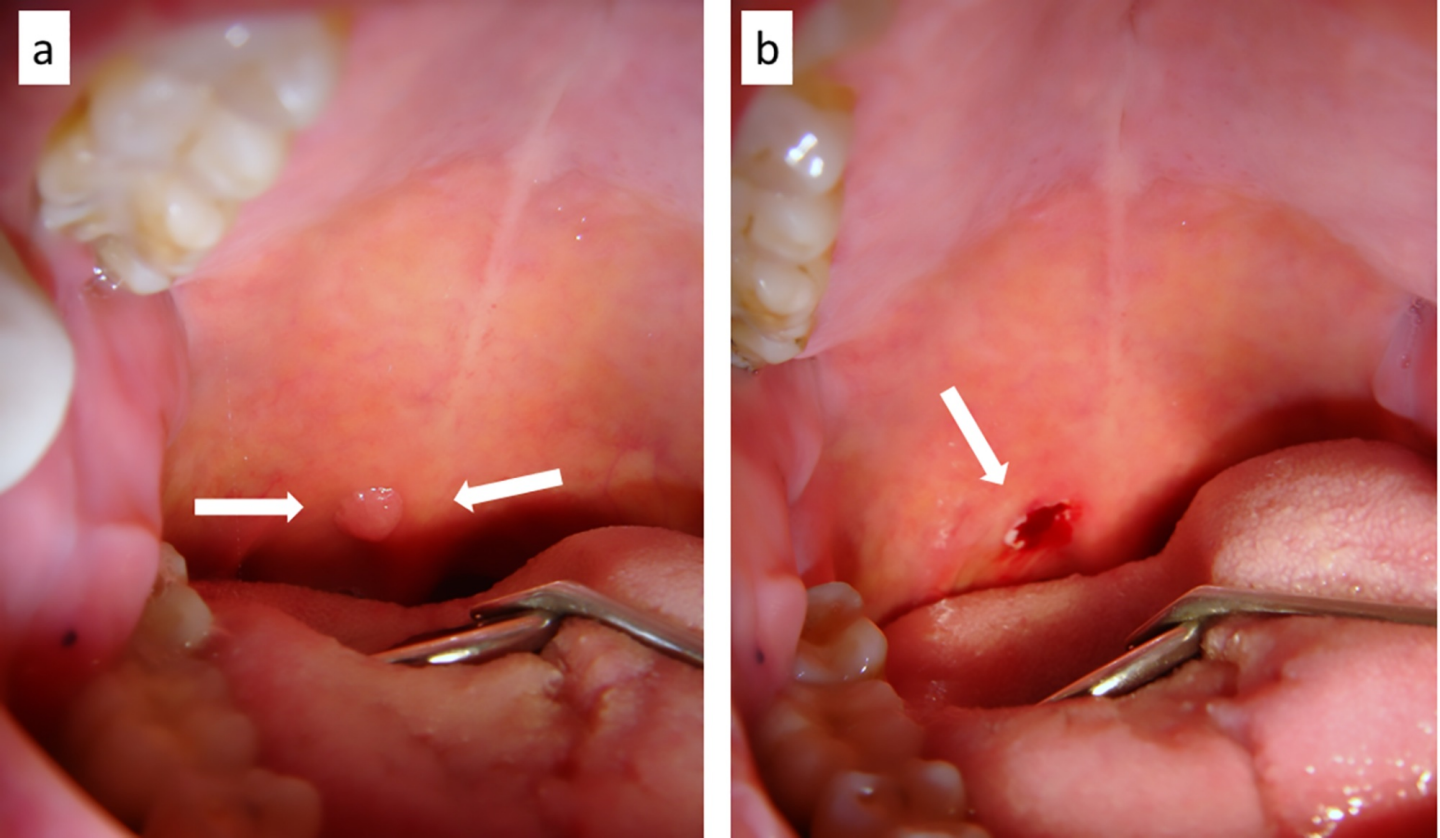
(a)Viral papilloma of the posterior soft palate (base of the uvula) (b) surgically removed by diode laser only with a small amount of local anesthesia

A 47-year-old man presented with a nodular lesion of the soft palate lasting six months (Figure 2a). More precisely, the lesion was sessile, painless, and located in the paramedian position at the base of the uvula. Because of the localization, and with the suspicion that it could be a proliferating viral lesion, the patient agreed to excision by DL under local anesthesia. The DL surgery (wavelength 980 ± 10 nm; continuous wave; fiber of 320 microns; output energy 1 W) was rapid, without bleeding (Figure 2b) or postoperative complications. The final diagnosis was that of viral papilloma, and no recurrence was detected at the one-year follow-up.

Case 3

**Figure 3:**
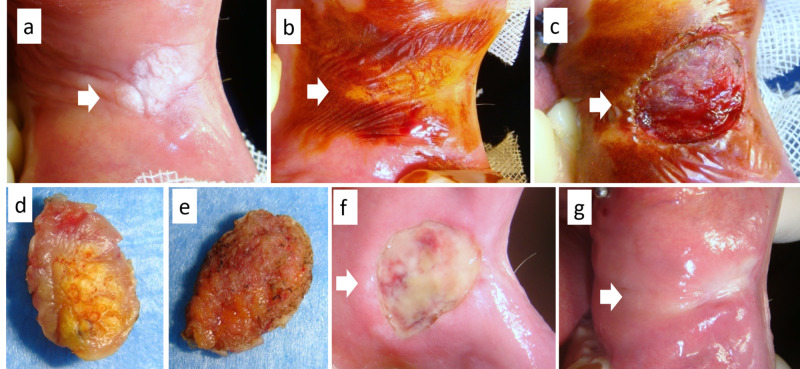
(a) Large dishomogeneous leukoplakia of the cheek in a smoker, (b) better defined by vital dye with Lugol’s solution, and diagnosed as frictional keratosis; (c) excision by diode laser without stitches and (d,e) the surgical sample; (f) partial healing after eight days and (g) complete mucosal healing after 18 days

The patient was a 58-year-old man who was a smoker and wore implant-retained prostheses. He experienced biting trauma for a long duration. Clinically, he exhibited a dishomogeneous leukoplakia of the left cheek (Figure 3a), whose margins were better defined by vital dye with Lugol’s iodine solution (Figure 3b). Considering the dimension and the impossibility of performing an incisional biopsy, the lesion was removed entirely by DL (wavelength 980 ± 10 nm; continuous wave; fiber of 320 microns; output energy 1.5 W; Figures 3c-3e) under light conscious sedation. Bleeding was absent during the procedure, stitches were unnecessary, and complete mucosal healing occurred in 18 days (Figures 3e-3g). The final diagnosis was of frictional keratosis.

Case 4

**Figure 4:**
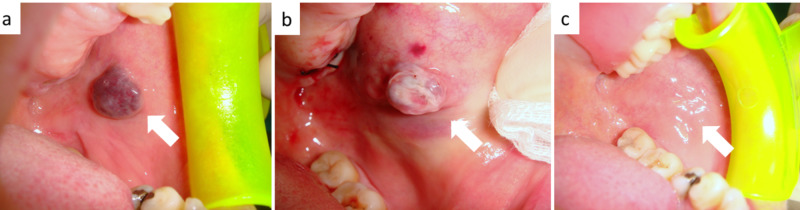
(a) Classic blue-violet appearance of a venous malformation of the cheeck mucosa; (b) transmucosal photocoagulation by diode laser confirmed by the color variation from blue-violet to grayish-white; (c) the irradiated area healed in 14 days

The patient was a 64-year-old man affected by a persistent nodular lesion of the cheek mucosa. More precisely, he presented with a blue-violet, sessile, painless, and nonbleeding lesion of the cheek lasting one year (Figure 4a). He reported occasional biting trauma. Warfarin therapy had been prescribed for the patient for chronic atrial fibrillation. The clinical diagnosis was of venous malformation, as confirmed by ultrasound investigation. Following a careful explanation of the treatment, the patient agreed to transmucosal photocoagulation by DL without suspension of warfarin treatment. After a small amount of local anesthesia, we used the DL (wavelength 910 ± 10 nm; pulsed-wave; fiber of 400 microns; output energy 5 W) to directly photocoagulate the lesion, and the treatment ended when its color varied from blue-violet to grayish-white (Figure 4b). The irradiated area healed in 14 days without recurrence (Figure 4c).

Case 5 

**Figure 5:**
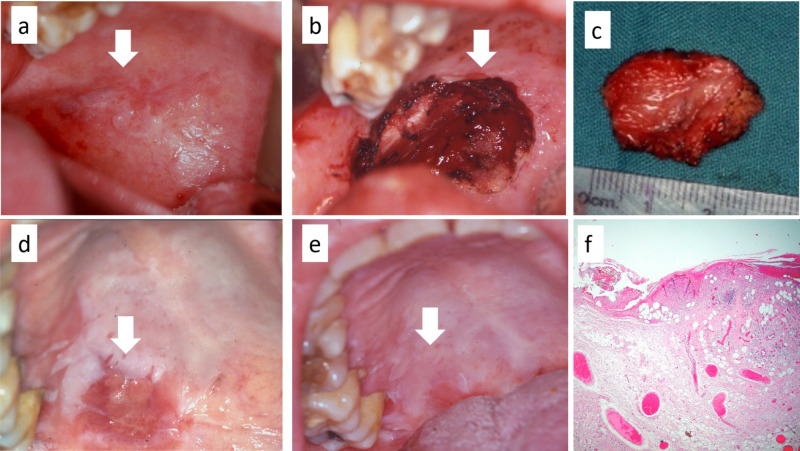
(a) Malignant transformation of proliferative verrucous leukoplakia of the soft palate; (b,c) diode laser excision of the entire lesion without direct suture of the margins; (d) partial and (e) complete healing after 22 days; (f) the histological examination shows foci of early oral squamous cell carcinoma

A 66-year-old woman affected by proliferative verrucous leukoplakia and who tested positive for the human hepatitis C virus exhibited a potentially malignant lesion of the soft palate at a routine clinical follow-up. Multiple nodular lesions arising within a dishomogeneous leukoplakia were observable on the soft palate in the paramedian position (Figure 5a). Considering the difficulties in treating the lesion with conventional scalpel surgery and the potential increase in bleeding related to the liver disease, a DL excision was performed (wavelength 980 ± 10 nm; continuous wave; fiber of 400 microns; output energy 2 W; Figures 5b, 5c) under general anesthesia. No intraoperative or postoperative bleeding occurred, and healing by second intention was completely achieved in 22 days (Figures 5d, 5e). The final diagnosis was early oral squamous cell carcinoma due to the malignant transformation of proliferative verrucous leukoplakia (Figure 5f). 

## Discussion

The cases in this series highlight several benefits of DL-assisted oral surgery, which include a decrease in the amount of local anesthesia required, absence of intraoperative bleeding, and precise and rapid surgery. Also, surgery using DL is associated with a lack of histological alterations to the surgical specimens if the DL is used within appropriate parameters. Additionally, stitches are rarely necessary with DL surgery, which allows for the possibility of removing large mucosal areas in difficult-to-access sites. DL surgery often has reduced postoperative bleeding complications, including edema and pain, accelerated mucosal rectification for second intention healing, reduced potential superinfection after surgery, and reduced or absent cosmetic sequela (especially for lesions occurring in the lip/lip vermilion) [1,2,4,8,9]. Furthermore, DL can be used to more easily manage patients on anticoagulant therapy that could not be safely discontinued for minor oral surgery, including photocoagulation of small venous malformations [12-14]. 

Second, intention healing is one of the most important clinical advantages of DL use for major oral surgery. It is frequently difficult to directly suture the surgical margins of large mucosal lesions or those occurring in unfavorable anatomical sites (e.g., soft palate, gingiva-buccal sulcus, retro-molar area in the mandible, and maxillary tuberosity) [2,6,8,11]. In such cases, DL surgery prevents more invasive procedures, such as additional flaps and skin grafts, for the direct coverage or closure of the surgical site. DL surgery also reduces the necessity of general anesthesia (for healthy and medically compromised patients), which can be safely replaced by conscious sedation in many cases [15-18]. However, direct suturing of the surgical margins, when possible, is always recommended after large excisions (e.g., for tongue lesions).

## Conclusions

This overview highlights the general benefits of DL-assisted surgery in the management of benign, premalignant, and malignant lesions of the oral cavity, as well as venous malformations. The achievable clinical advantages are essentially related to the intrinsic properties of the DL wavelength that, if correctly used, may simplify all procedures of minor and major oral surgery. With DL use, the need for general anesthesia certainly decreases for the treatment of oral lesions with wide dimensions or those occurring in uncomfortable anatomical sites or uncooperative patients/children, and more frequently, may be replaced by easily manageable conscious sedation.

## References

[1] Luke AM, Mathew S, Altawash MM, Madan BM (2019). Lasers: a review with their applications in oral medicine. J Lasers Med Sci.

[2] Romanos G, Nentwig GH (1999). Diode laser (980 nm) in oral and maxillofacial surgical procedures: clinical observations based on clinical applications. J Clin Laser Med Surg.

[3] Limongelli L, Tempesta A, De Caro A, Maiorano E, Angelelli G, Capodiferro S, Favia G (2019). Diode laser photocoagulation of intraoral and perioral venous malformations after tridimensional staging by high-definition ultrasonography. Photobiomodul Photomed Laser Surg.

[4] Derikvand N, Chinipardaz Z, Ghasemi S, Chiniforush N (2016). The versatility of 980 nm diode laser in dentistry: a case series. J Lasers Med Sci.

[5] Capodiferro S, Limongelli L, Tempesta A, Maiorano E, Favia G (2018). Diode laser treatment of venous lake of the lip. Clin Case Rep.

[6] Aldelaimi TN, Khalil AA (2015). Clinical application of diode laser (980 nm) in maxillofacial surgical procedures. J Craniofac Surg.

[7] Angiero F, Parma L, Crippa R, Benedicenti S (2012). Diode laser (808 nm) applied to oral soft tissue lesions: a retrospective study to assess histopathological diagnosis and evaluate physical damage. Lasers Med Sci.

[8] Tachmatzidis T, Dabarakis N (2016). Technology of lasers and their applications in oral surgery: literature review. Balk J Dent Med.

[9] De Falco D, Di Venere D, Maiorano E (2020). Diode laser surgery of recurrent white lesion of the lip: clinicopathological consideration and cosmetic outcome. Cureus.

[10] Capodiferro S, Tempesta A, Limongelli L, Maiorano E, Benedicenti S, Favia G (2019). Nonsurgical periodontal treatment by erbium:YAG laser promotes regression of gingival overgrowth in patient taking cyclosporine A: a case report. Photobiomodul Photomed Laser Surg.

[11] Limongelli L, Capodiferro S, Tempesta A (2020). Early tongue carcinomas (clinical stage I and II): echo-guided three-dimensional diode laser mini-invasive surgery with evaluation of histological prognostic parameters. A study of 85 cases with prolonged follow-up. Lasers Med Sci.

[12] Bacci C, Sacchetto L, Zanette G, Sivolella S (2018). Diode laser to treat small oral vascular malformations: a prospective case series study. Lasers Surg Med.

[13] António N, Castro G, Ramos D, Machado A, Gonçalves L, Macedo T, Providência LA (2008). The debate concerning oral anticoagulation: whether to suspend oral anticoagulants during dental treatment. (Article in Portuguese). Rev Port Cardiol.

[14] Isola G, Matarese G, Cordasco G, Rotondo F, Crupi A, Ramaglia L (2015). Anticoagulant therapy in patients undergoing dental interventions: a critical review of the literature and current perspectives. Minerva Stomatol.

[15] Dell'Olio F, Capodiferro S, Lorusso P (2019). Light conscious sedation in patients with previous acute myocardial infarction needing exodontia: an observational study. Cureus.

[16] Flick W, Lloyd M (2019). Illinois dental anesthesia and sedation survey for 2016. Anesth Prog.

[17] Fiorillo L (2019). Conscious sedation in dentistry. Medicina.

[18] Coulthard P (2019). Medical management of dental anxiety. Prim Dent J.

